# CD19^+^CD24^hi^CD38^hi^ B Cell Dysfunction in Primary Biliary Cholangitis

**DOI:** 10.1155/2020/3019378

**Published:** 2020-02-10

**Authors:** Qubo Chen, Lanmin Lai, Xiaoling Chi, Xinyi Lu, Huaxian Wu, Jing Sun, Weilin Wu, Li Cai, Xuan Zeng, Chuyang Wang, WeiCheng Chen, Anping Peng

**Affiliations:** ^1^Biological Resource Center, The Second Affiliated Hospital of Guangzhou University of Chinese Medicine, 510120 Guangzhou, China; ^2^Division of Hepatology, The Second Affiliated Hospital of Guangzhou University of Chinese Medicine, 510120 Guangzhou, China; ^3^Department of Laboratory Science, The Second Affiliated Hospital of Nanfang Medical University, 510120 Guangzhou, China

## Abstract

CD19^+^CD24^hi^CD38^hi^ B cells are immature transitional B cells that, in normal individuals, exert suppressive effects by IL-10 production but are quantitatively altered and/or functionally impaired in individuals with various autoimmune diseases. Primary biliary cholangitis (PBC), an autoimmune disease, clinically presents as chronic cholestasis and nonsuppurative destructive cholangitis. A role for CD19^+^CD24^hi^CD38^hi^ B cells in PBC is unknown. This study investigated the frequency and functional variation of circulating CD19^+^CD24^hi^CD38^hi^ B cells in PBC patients. Flow cytometry was employed to quantify the percentage of CD19^+^CD24^hi^CD38^hi^ B cells in peripheral blood samples. Correlations between CD19^+^CD24^hi^CD38^hi^ B cells and routine laboratory parameters were assessed. Levels of IL-10, TNF-*α*, IL-6 and IL-12, and Tim-1 in CD19^+^CD24^hi^CD38^hi^ B cells from PBC patients were analyzed. The effect of CD19^+^CD24^hi^CD38^hi^ B cells on CD4^+^T cell differentiation was evaluated. The percentage of CD19^+^CD24^hi^CD38^hi^ B cells in PBC patients was significantly higher than in healthy controls and was positively correlated with liver cholestasis. After activation by anti-B cell receptor and CpG, the production of IL-10 was decreased and the production of IL-6 and IL-12 was increased in CD19^+^CD24^hi^CD38^hi^ B cells from PBC patients. Moreover, Tim-1 levels were significantly downregulated in CD19^+^CD24^hi^CD38^hi^ B cells from PBC patients. Coculture showed that PBC-derived CD19^+^CD24^hi^CD38^hi^ B cells were less capable of CD4^+^T cell inhibition, but promoted Th1 cell differentiation. In conclusion, PBC patients have expanded percentages, but impaired CD19^+^CD24^hi^CD38^hi^ B cells, which correlate with disease damage. In PBC patients, this B cell subset has a skewed proinflammatory cytokine profile and a decreased capacity to suppress immune function, which may contribute to the pathogenesis of PBC.

## 1. Introduction

Primary biliary cholangitis (PBC) is a chronic autoimmune liver disease that is characterized by a gradual loss of small bile ducts. The pathogenesis of PBC is not well understood. Both autoimmune and inflammatory mechanisms have been described for PBC [1]. Despite high levels of antimitochondrial antibody (AMA), high levels of serum IgM, and the presence of infiltrating B cells in liver portal areas of PBC patients, a role for B cells in PBC has not been well identified [2–5]. Previous investigations have primarily concentrated on autoantibody-producing B cells, with few investigations of regulatory B cells (Breg).

In addition to producing antibodies and acting as important antigen-presenting cells for T cell priming and cytokine production, B cells also play a negative role during immune responses [6, 7]. Breg contribute to the maintenance of peripheral immune tolerance and tissue protection by downmodulation of T and B cell function [8]. Due to a lack of distinct cell surface markers, no Breg-specific transcription factors, and a lack of a unique cell lineage, investigation of Breg has been hampered [9]. In many conditions, Breg function is dependent upon IL-10 [9]. Lack or loss of IL-10-producing Breg exacerbates many autoimmune and inflammatory diseases, including arthritis, lupus, chronic colitis, and experimental autoimmune encephalomyelitis [10–15]. In addition to IL-10, Breg also produce proinflammatory cytokines, such as TNF-*α* and IL-6, which also participate in Breg function [16, 17]. A combination of IL-10 and proinflammatory cytokines may best describe Breg function.

T cell immunoglobulin mucin domain-1 (Tim-1), a transmembrane glycoprotein, was recently identified as an inclusive marker for IL-10^+^Breg [18, 19]. More importantly, Tim-1 plays a key role in Breg function by maintaining immune tolerance [20, 21]. A Tim-1 mutant mouse has a defect in B cell-derived IL-10 production [19]. Tim-1 defects also increase proinflammatory cytokine production, including IL-1, IL-6, and TNF-*α* [20]. Further, Tim-1-deficient B cells enhance Th1 and Th17 cell responses and inhibit regulatory T cells [20, 22].

For phenotypic identification of human Breg, CD19^+^CD24^hi^CD38^hi^ B cells [10, 11, 14, 16, 23, 24] and CD19^+^CD5^+^CD1d^hi^ B cells [25–27] have been used. The majority of CD19^+^CD5^+^CD1d^hi^ B cells are found within CD19^+^CD24^hi^CD38^hi^ B cells [14]. CD19^+^CD24^hi^CD38^hi^ B cells, also known as immature/transitional B cells, contain a high proportion of IL-10-producing cells [10, 16, 23] that act to suppress CD4^+^T responses [10, 14]. Altered numbers and/or function of CD19^+^CD24^hi^CD38^hi^ B cells are associated with the pathogenesis of various autoimmune diseases, including systemic lupus erythematosus (SLE) [10, 15], rheumatoid arthritis (RA) [14], ulcerative colitis [11], and ankylosing spondylitis [24].

In this study, we analyzed CD19^+^CD24^hi^CD38^hi^ B cells from peripheral blood of PBC patients to investigate the frequency, proliferative capacity and cytokine production of circulating CD19^+^CD24^hi^CD38^hi^ B cells, and the regulatory capacity of this B cell subset in PBC, in order to explore the role of CD19^+^CD24^hi^CD38^hi^ B cells in the pathogenesis of PBC.

## 2. Materials and Methods

### 2.1. Patients and Specimens

Fresh EDTA anticoagulated peripheral blood samples were obtained from patients diagnosed with PBC (*n* = 38) and from healthy control subjects (HC, *n* = 38). PBC patients were diagnosed according to the Clinical Practice Guidelines by the European Association for the Study of the Liver (EASL) [28]: all individuals were subjected to the elevated serum alkaline phosphatase (ALP) and the presence of antimitochondrial antibody (AMA and AMA type M_2_) in serum. Patients who had other autoimmune diseases such as diabetes, chronic colitis, arthritis, and lupus and other liver diseases including alcoholic liver disease, nonalcoholic fatty liver diseases, hepatitis B and C, and autoimmune hepatitis were excluded from this study. Clinical and biochemical data for the PBC patients are found in Table 1. According to this, age of patients, number of case, and the dominance of female gender were well matched between PBC patients and enrolled healthy controls. The research protocol was approved by the Second Affiliated Hospital of Guangzhou University of Chinese Medicine Research Ethnics Committee, and written informed consent was received from all participants.

### 2.2. PBMC Isolation, Storage, and Culture

Peripheral blood mononuclear cells (PBMC) were isolated by density centrifugation using Ficoll-Paque (GE Healthcare). PBMC were stored in 90% fetal calf serum (FCS) (Bio Tech) and 10% DMSO (Sigma) and cryopreserved in liquid nitrogen until evaluation. Cells were cultured in completed RPMI 1640 supplemented with 10% FCS (HyClone, GE Healthcare, USA) and 1% penicillin/streptomycin.

### 2.3. Flow Cytometry and Cell Sorting

Flow cytometry was performed with the following antibodies: anti-CD19-Percp-Cy5.5, anti-CD19-Percp-eFluor710, anti-CD24-FITC, anti-CD38-superbright600, anti-CD38-PE, anti-Ki-67-Alexa Fluor647, anti-IL-10-PE, anti-TIM-1-PE, anti-CD4-FITC, anti-IFN-*γ*-PE, anti-IL-17A-APC (eBioscience, San Diego CA, USA), anti-IL-6-APC, anti-TNF-*α*-FITC, anti-IL-12-BV-421, and anti-CD24-Alexa Fluor647 (BD Biosciences, France). Intracellular cytokines and Ki-67 were assessed in cells treated with Permeabilization and IC Fixation Buffers (eBioscience, San Diego, CA, USA). Stained cells were analyzed with a 21-color ZE5 cell analyzer (Bio-Rad, USA). The CD19^+^CD24^hi^CD38^hi^ B subset was sorted (FACSAria; BD Pharmingen) using anti-CD19-Percp-Cy5.5, anti-CD24-FITC, and anti-CD38-PE. The sort purity of CD19^+^CD24^hi^CD38^hi^ B cells was routinely >90%. CD4^+^T subpopulation was sorted (FACSAria; BD Pharmingen) using anti-CD38-PE and anti-CD3-PE. The sort purity of CD4^+^T cells was routinely >95%. All flow cytometry data were analyzed with FlowjoV10 Software (Tree Star, OR, USA).

### 2.4. B Cell Activation

PBMC were cultured for 48 h in RPMI 1640 medium supplemented with 10% FCS (HyClone, GE Healthcare, USA) at a density of 2 × 10^6^ cells/mL. Cells were also cultured in the presence or absence of 5 *μ*g/mL polyclonal anti-human IgA+IgG+IgM (H+L) goat antibodies (Jackson Immunoresearch, West Grove, PA, USA) for B cell receptor (BCR) activation and 3 *μ*g/mL ODN 2006 CpG oligonucleotide for toll-like receptor (TLR9) (Invivogen, San Diego, CA, USA) activation. During the last 6 h of culture, cells were stimulated with 50 ng/mL phorbol 12-myristate 13-acetate (PMA), 1 *μ*g/mL ionomycin, and 1 *μ*g/mL Brefeldin A (BioLegend, San Diego, CA, USA). Intracellular detection of cytokines in CD19^+^CD24^hi^CD38^hi^ B cells included IL-10, IL-6, and TNF-*α* using a ZE5 cell analyzer (Bio-Rad, USA).

### 2.5. CD4^+^T Cell and CD19^+^CD24^hi^CD38^hi^ B Cell Coculture

Healthy control (HC) CD4^+^T cells were mixed with autologous or PBC CD19^+^CD24^hi^CD38^hi^ B cells at a ratio of 5 : 1 in culture medium containing RPMI 1640 (HyClone, Logan, UT, USA) supplemented with 10% fetal bovine serum (Gibco, Carlsbad, CA) and 1% penicillin/streptomycin (Invitrogen, Carlsbad, CA). The cells were stimulated with 1 *μ*g/mL plate-bound anti-CD3 antibody (Invitrogen, Carlsbad, CA) for 6 days, and cells were stimulated with 50 ng/mL PMA, 1 *μ*g/mL ionomycin, and 1 *μ*g/mL Brefeldin A (BioLegend, San Diego, CA, USA) for the last 6 h of culture to test for the production of intracellular IFN-r in CD4^+^T cells.

### 2.6. Statistical Analysis

Data were analyzed using GraphPad Prism 6 and presented as the mean ± SD. Unpaired Student's *t*-test was used to assess significance between two groups. One-way analysis of variance (ANOVA) was used to assess significant differences between group means (≥3 groups), and Bonferroni corrections were applied for ANOVA. Pearson's/Spearman's correlation coefficients were used to assess correlations. *P* values < 0.05 were considered to be statistically significant.

## 3. Results

### 3.1. PBC Patients Exhibit Enhanced Frequency of Peripheral Blood CD19^+^CD24^hi^CD38^hi^ B Cells

**Figure 1:**
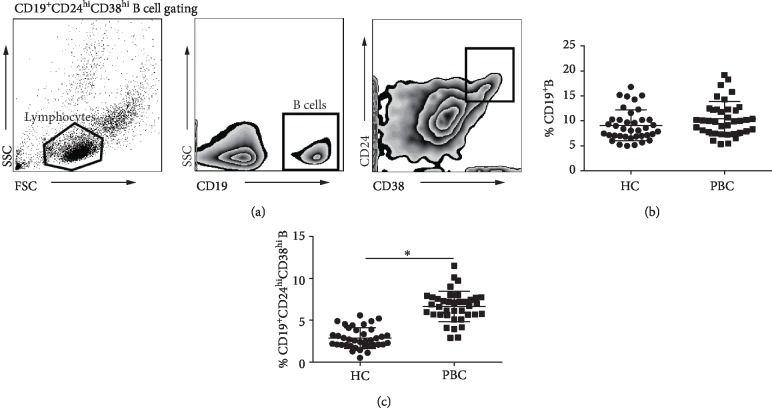
Frequencies of CD19^+^CD24^hi^CD38^hi^ B cells were upregulated in the peripheral blood of PBC patients. (a) The gating strategy for CD19^+^CD24^hi^CD38^hi^ B cells by flow cytometry. (b) Statistical analysis of circulating CD19^+^ B cell frequencies in PBC patients and HC subjects. (c) Statistical analysis of circulating CD19^+^CD24^hi^CD38^hi^ B cell frequencies in PBC patients and HC subjects; ^∗^*P* < 0.05.

PBMC were phenotypically analyzed using flow cytometry for the levels of surface markers, including CD19, CD24, and CD38. B cells were defined as CD19^+^ lymphocytes. Within the CD19^+^ B cell gate, CD24^hi^CD38^hi^ cells were defined as CD19^+^CD24^hi^CD38^hi^ B cells. The gating strategy is illustrated by representative flow cytometric dots (Figure 1(a)). In agreement with previous results [29], there was no statistical difference in the percentage of circulating CD19^+^ B cells between PBC patients and HC subjects (Figure 1(b)). As shown in Figure 1(c), we observed a significant increase in the frequency of circulating CD19^+^CD24^hi^CD38^hi^ B cells in PBC patients, compared to the control group.

### 3.2. CD19^+^CD24^hi^CD38^hi^ B Cells Are Highly Proliferative in PBC Patients

**Figure 2:**
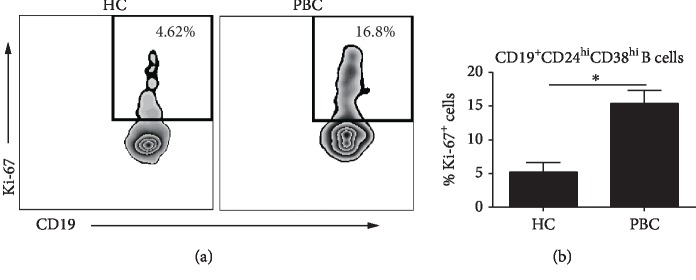
CD19^+^CD24^hi^CD38^hi^ B cells were highly proliferative from PBC patients compared to HC subjects. PBMC samples were stained with B cell surface markers CD19, CD24, and CD38. Intranuclear marker of proliferation was Ki-67. (a) Representative flow cytometric dot plots of Ki-67 expression in CD19^+^CD24^hi^CD38^hi^ B cells from PBC patients and HC subjects. (b) The frequency of Ki-67^+^ B cells summarized for CD19^+^CD24^hi^CD38^hi^ B cells from PBC patients and HC subjects; ^∗^*P* < 0.05.

Since CD19^+^CD24^hi^CD38^hi^ B cells were significantly upregulated in PBC patients, the proliferation capacity of these cells was investigated. Antigen Ki-67 is a nuclear protein expressed in proliferating mammalian cells and generally identifies cell proliferation. PBMC were assessed for Ki-67 staining using flow cytometry. Ki-67 staining was significantly increased in CD19^+^CD24^hi^CD38^hi^ B cells from PBC patients, compared to HC subjects (Figures 2(a) and 2(b)). CFSE staining also showed that CD19^+^CD24^hi^CD38^hi^ B cell proliferation from PBC patients was upregulated, compared to HC subjects (Supplementary [Supplementary-material supplementary-material-1]).

### 3.3. Elevated CD19^+^CD24^hi^CD38^hi^ B Cells Correlated with TBIL, DBIL, and TBA in PBC Patients

**Figure 3:**
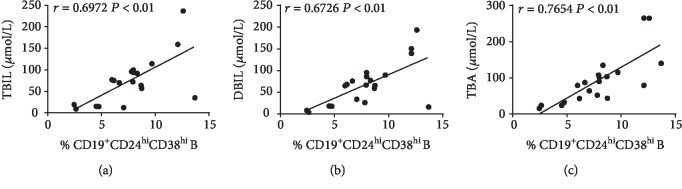
Correlations between CD19^+^CD24^hi^CD38^hi^ B cell frequencies and different laboratory parameters. (a) Significant positive correlations were found between levels of serum TBIL and the percentage of circulating CD19^+^CD24^hi^CD38^hi^ cells in PBC patients. (b) Significant positive correlations were found between levels of serum DBIL and the percentage of circulating CD19^+^CD24^hi^CD38^hi^ cells in PBC patients. (c) Significant positive correlations were found between levels of serum TBA and the percentage of circulating CD19^+^CD24^hi^CD38^hi^cells in PBC patients; ^∗^*P* < 0.05.

To evaluate the clinical significance of the CD19^+^CD24^hi^CD38^hi^ B cells in PBC patients, we examined possible correlations between CD19^+^CD24^hi^CD38^hi^ B cell frequencies and laboratory parameters. As shown in Figure 3, there was a correlation between the frequency of circulating CD19^+^CD24^hi^CD38^hi^ B cells and TBIL (*r* = 0.6972, *P* < 0.01), DBIL (*r* = 0.6726, *P* < 0.01), and TBA (*r* = 0.7654, *P* < 0.01). However, no significant correlations were found between the frequency of circulating CD19^+^CD24^hi^CD38^hi^ B cells and ALP, GGT, AST, and ALT levels (data were not shown). Circulating CD19^+^CD24^hi^CD38^hi^ B subset frequency did not differ in whole disease stage (Supplementary [Supplementary-material supplementary-material-1]).

### 3.4. IL-10 Production Is Decreased in Activated PBC CD19^+^CD24^hi^CD38^hi^ B Cells

**Figure 4:**
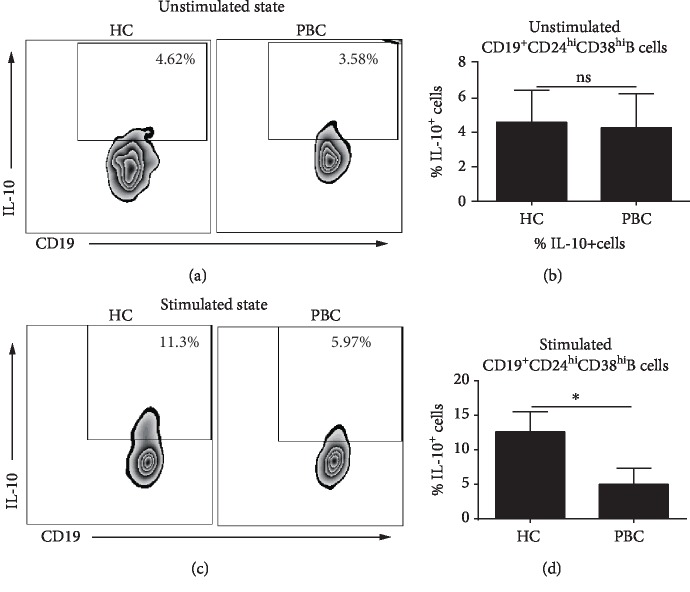
CD19^+^CD24^hi^CD38^hi^ B cells from PBC patients express lower level of IL-10 when stimulated by anti-BCR and CpG, compared to HC subjects. PBMC from PBC and healthy individuals were stimulated for 48 h with or without anti-BCR and CpG. Phorbol ester, plus ionomycin, plus Brefeldin A were added for the last 5 h of culture. Cells were surface stained with CD19, CD24, and CD38 monoclonal antibodies (mAbs) and intracellularly stained for IL-10. (a, c) Representative intracellular IL-10 staining in unstimulated or stimulated CD19^+^CD24^hi^CD38^hi^ B cells in the peripheral blood of one PBC patient and healthy control. (b, d) Statistical graph of the frequency of IL-10^+^ cells in unstimulated or stimulated CD19^+^CD24^hi^CD38^hi^ B cells from PBC patients and HC subjects; ^∗^*P* < 0.05.

CD19^+^CD24^hi^CD38^hi^ B cells are the principal IL-10-expressing B subset, so IL-10 expression in the CD19^+^CD24^hi^CD38^hi^ B subset was compared for PBC patients and HC subjects by intracellular staining. Without stimulation, no significant difference in IL-10 expression by CD19^+^CD24^hi^CD38^hi^ B cells was observed between PBC patients and HC subject (Figures 4(a) and 4(b)). Combined stimulation of the BCR and TLR9 in human B cells and Breg induces robust IL-10 secretion [30, 31]. Thus, CD19^+^CD24^hi^ CD38^hi^ B subsets from PBC patients and HC subjects were activated with CpG and anti-BCR for 48 h and analyzed for IL-10 expression by intracellular staining. As shown in Figures 4(c) and 4(d), the percentage of IL-10^+^CD19^+^CD24^hi^CD38^hi^ B cells was 13.36 ± 1.88% in HC subjects and 5.56 ± 1.25% in PBC patients. PBC patients had significantly decreased IL-10 levels in CD19^+^CD24^hi^CD38^hi^ B cells upon activation with CpG and anti-BCR. CD19^+^CD24^hi^CD38^hi^ B cells from HC responded well to CpG and anti-BCR combined stimulation and increased their IL-10^+^ transitional B cell percentage contrarily to B cells from PBC patients who kept similar frequency.

### 3.5. IL-6 and IL-12 Expression Is Increased in Activated CD19^+^CD24^hi^CD38^hi^ B Cells from PBC Patients

**Figure 5:**
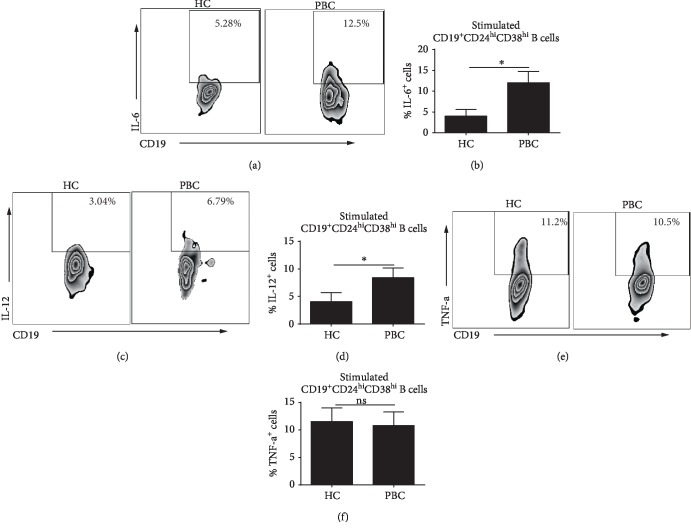
CD19^+^CD24^hi^CD38^hi^ B cells from PBC patients express greater amounts of IL-6 and IL-12 when stimulated with anti-BCR and CpG. PBMC were stimulated with CpG and anti-BCR for 48 h plus phorbol ester, ionomycin, and Brefeldin A (last 5 h). (a, c, e) Each representative dot plot shows the frequency of IL-6, IL-12, or TNF-*α* within the CD19^+^CD24^hi^CD38^hi^ B subset from PBC or HC. (b, d, f) Cumulative frequency of IL-6-, IL-12-, or TNF-*α*-producing cells within the CD19^+^CD24^hi^CD38^hi^ B subsets from PBC patients or HC subjects; ^∗^*P* < 0.05.

The coexpression of the proinflammatory cytokines IL-6, TNF-*α*, and IL-12 was assessed in CD19^+^CD24^hi^CD38^hi^ B cells from PBC patients after activation with CpG and anti-BCR. PBMC were stimulated with CpG and anti-BCR for 48 h, and cytokines were detected by intracellular staining. Compared to HC subjects, PBC patients exhibited a markedly increased expression of IL-6 (Figures 5(a) and 5(b)) and IL-12 (Figures 5(c) and 5(d)) in stimulated CD19^+^CD24^hi^CD38^hi^ B cells. The expression of TNF-*α* was unchanged (Figures 5(e) and 5(f)).

### 3.6. Tim-1 Expression Is Downregulated in CD19^+^CD24^hi^CD38^hi^ B Cells from PBC Patients

**Figure 6:**
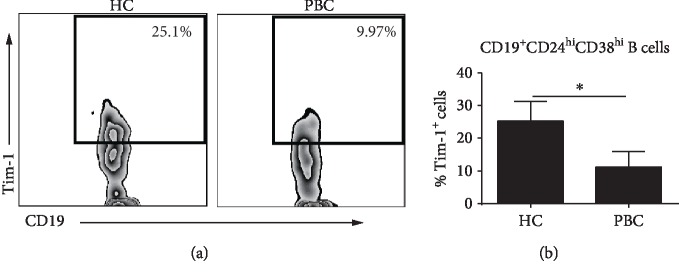
Tim-1 expression in CD19^+^CD24^hi^CD38^hi^ B cells was reduced in PBC patients. (a) Representative flow cytometric dot plots show the frequency of Tim-1^+^ cells in CD19^+^CD24^hi^CD38^hi^ B cells from PBC patients and HC subjects. (b) Statistical graph of the frequency of Tim-1^+^ cells in CD19^+^CD24^hi^CD38^hi^ B cells from PBC patients and HC subjects; ^∗^*P* < 0.05.

Tim-1 expression and signaling are essential for the maintenance and promotion of IL-10 production in CD19^+^CD24^hi^CD38^hi^ B cells. Moreover, a Tim-1 defect in CD19^+^CD24^hi^CD38^hi^ B cells alters the balance between regulatory and proinflammatory cytokines. The expression of Tim-1 in CD19^+^CD24^hi^CD38^hi^ B cells from PBC patients was assessed by flow cytometry. The Tim-1-expressing cell frequency was approximately 25% in CD19^+^CD24^hi^CD38^hi^ B cells from HC subjects, while PBC patients exhibited a markedly decreased frequency of Tim-1-expressing cells (approximately 12%) (Figures 6(a) and 6(b)).

### 3.7. CD19^+^CD24^hi^CD38^hi^ B Cells from PBC Patients Promote the Differentiation of CD4^+^T Cells into Th1 Cells

**Figure 7:**
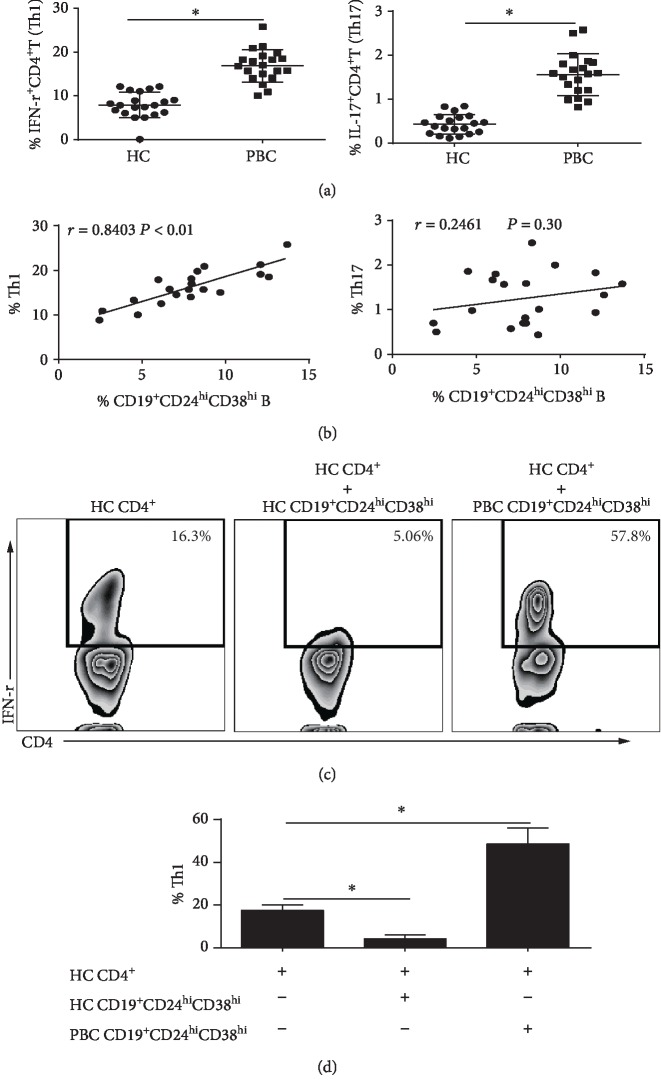
CD19^+^CD24^hi^CD38^hi^ B cells from PBC patients failed to suppress Th1 cell differentiation, but promoted Th1 cell differentiation. PBMC isolated from PBC patients or HC subjects were divided into two populations. One was stained with CD19, CD24, and CD38 mAbs and assessed by flow cytometry. The other was stimulated with phorbol ester, ionomycin, and Brefeldin A for 6 h, then surface stained with CD4 mAbs, permeabilized, and stained with IFN-*γ* or IL-17 mAbs and assessed by flow cytometry. (a) Graphs showing the frequency of Th1 and Th17 cells from 20 PBC patients and 20 HC subjects. (b) Correlation between CD19^+^CD24^hi^CD38^hi^ B cell frequency and Th1 or Th17 cell frequency from PBC patients. (c, d) CD19^+^CD24^hi^CD38^hi^ B cells from PBC patients and HC subjects were sorted by flow cytometry and then cocultured with HC subject subset CD^+^T cells for 6 D, and Th1 cell frequency was measured by flow cytometry. Representative (c) contour plots and (d) graphs showed healthy CD19^+^CD24^hi^CD38^hi^ B cells suppressed autologous Th1 cell differentiation, while PBC CD19^+^CD24^hi^CD38^hi^ B cells promoted HC Th1 cell differentiation. The data are representative of four separate experiments; ^∗^*P* < 0.05.

IFN-*γ*^+^CD4^+^T cells (Th1) and IL-17A^+^CD4^+^T cells (Th17) have been associated with the pathogenesis of PBC. Intracellular cytokine staining test demonstrated that the frequencies of peripheral Th1 and Th17 cells were upregulated (Figure 7(a)) in PBC patients, as previously reported [32, 33]. Since CD19^+^CD24^hi^CD38^hi^ B cells are important IL-10-producing B cells that may be suppressive, we assessed whether this subset isolated from PBC patients prevented the differentiation of CD4^+^T cells. First, we investigated the possible correlation between CD19^+^CD24^hi^CD38^hi^ B cells and Th1 and Th17 cells.  Figure 7(b) demonstrates the CD19^+^CD24^hi^CD38^hi^ B cell frequency to be positively correlated with Th1 cell frequency (*r* = 0.8403, *P* < 0.01), but not with Th17 cell frequency (*r* = 0.2461, *P* = 0.30). Sorted CD4^+^T cells from HC subjects were cocultured with autologous CD19^+^CD24^hi^CD38^hi^ B cells from HC subjects or allogeneic CD19^+^CD24^hi^CD38^hi^ B cells from PBC patients. Coculture of CD19^+^CD24^hi^CD38^hi^ B cells isolated from HC subjects with autologous CD4^+^T cells resulted in a significant decrease in Th1 cell differentiation when compared to CD4^+^T cells cultured alone (Figures 7(c) and 7(d)), as reported previously [10]. Interestingly, CD19^+^CD24^hi^CD38^hi^ B cells from PBC patients lacked a similar degree of suppressive capacity, but did promote Th1 cell differentiation (Figures 7(c) and 7(d)). In addition, coculture of CD19^+^CD24^hi^CD38^hi^ B cells isolated from HC subjects with PBC CD4^+^T cells also resulted in a significant decrease in Th1 cell differentiation, while coculture of CD19^+^CD24^hi^CD38^hi^ B cells isolated from PBC subjects with autologous CD4^+^T cells also resulted in a significant increase in Th1 cell differentiation (Supplementary [Supplementary-material supplementary-material-1]).

## 4. Discussion and Conclusions

In this study, an expansion of immature transitional B cells (CD19^+^CD24^hi^CD38^hi^ B cells) was demonstrated as well as altered function for this subset in the circulation of PBC patients. The evidence is as follows. First, CD19^+^CD24^hi^CD38^hi^ B cells from PBC patients had a skewed proinflammatory cytokine phenotype, with decreased IL-10 and increased of IL-6 and IL-12 production, when activated with CpG and anti-BCR. Second, Tim-1 expression was defective in this subset. Finally, this B cell subpopulation failed to prevent the CD4^+^T cell differentiation into Th1, but rather promoted Th1 cell differentiation.

CD19^+^CD24^hi^CD38^hi^ B cells from PBC patients proliferated and underwent more cell division *in vivo* than cells from HC subjects. This result suggests this subset to be potentially linked to disease or alternatively to reflect chronic immune system stimulation. To assess the potential link between this subset and disease, CD19^+^CD24^hi^CD38^hi^ B cells were correlated with clinical liver function indicators and clinical stages in PBC patients. The results showed that the frequency of this subpopulation positively correlated with TBIL, DBIL, and TBA. Bilirubin is an important indicator of liver metabolism. Metabolites such as TBIL and DBIL serve as reference values for the degree of liver cholestasis [34]. Serum TBA not only reflects the degree of liver parenchymal damage but also indicates abnormalities in the intrahepatic bile blood barrier [35]. In addition, TBA has a high degree of sensitivity for the diagnosis of bile duct obstruction and intrahepatic cholestasis [36]. These results suggest that CD19^+^CD24^hi^CD38^hi^ B cells may be involved in the immune pathogenesis of PBC. Although the frequency of CD19^+^CD24^hi^CD38^hi^ B cells was similar regardless of the stage of the disease, the regulatory function of this subset might be differently affected. It is therefore necessary to further study the regulation mediated by this subgroup between PBC patients with different clinical stages to understand whether any CD19^+^CD24^hi^CD38^hi^ B cell dysfunction participates in the severity of the disease.

Defects in regulatory CD19^+^CD24^hi^CD38^hi^ B cells have been identified in several autoimmune diseases, including SLE [10], RA [14], ulcerative colitis [11], ankylosing spondylitis [24], and juvenile dermatomyositis [16]. Important to the suppressive capacity of human CD19^+^CD24^hi^CD38^hi^ B cells is IL-10 [10, 14]. In SLE patients, CD19^+^CD24^hi^CD38^hi^ B cells are defective in IL-10 expression and are unable to inhibit Th1 cell responses *in vitro* [10, 15, 35]. In active RA patients, CD19^+^CD24^hi^CD38^hi^ B cells restrict the differentiation of CD4^+^T cells into Th1 cells, but not into Th17 cells [14]. We did not observe a difference in unstimulated CD19^+^CD24^hi^CD38^hi^ B cell IL-10 production between PBC patients and controls. CD19^+^CD24^hi^CD38^hi^ B cells from PBC patients display impaired IL-10 production when activated, which suggests that CD19^+^CD24^hi^CD38^hi^ B cells from PBC patients are defective in their production of IL-10. CD19^+^CD24^hi^CD38^hi^ B cells are known to produce proinflammatory cytokines TNF-*α* and IL-6 [16, 17]. Results herein showed that CD19^+^CD24^hi^CD38^hi^ B cells from PBC patients displayed increased IL-6 and IL-12 production when activated with anti-BCR and CpG. These results indicated that for different clinical or pathologic states, the cytokine profile of CD19^+^CD24^hi^CD38^hi^ B cells can change and skew toward a proinflammatory profile. Compared to IL-10 alone, measurement of IL-10 and proinflammatory cytokines may be a better predictor of regulatory B activity.

Tim-1 signaling is essential for the maintenance and promotion of IL-10 production by CD19^+^CD24^hi^CD38^hi^ B cells. Moreover, Tim-1 defects alter the balance between anti-inflammatory and proinflammatory cytokine expression within CD19^+^CD24^hi^CD38^hi^ B cells and other specific B cell subsets [19–21]. The majority of Tim^+^CD19^+^CD24^hi^CD38^hi^ B cells also coexpress IL-10. PBC patients exhibited a significant decrease in Tim^+^CD19^+^CD24^hi^CD38^hi^ B cells, which suggests that cytokine polarization may be correlated with lower levels of Tim-1, although other possibilities cannot be excluded.

In healthy humans, peripheral CD19^+^CD24^hi^CD38^hi^ B cells are known to negatively regulate immunity and to inhibit Th1 cell proliferation and differentiation, which maintains immune balance. Effects of the subset on Th17 cell proliferation and differentiation are controversial [10, 14]. Our results showed that CD19^+^CD24^hi^CD38^hi^ B cells from HC subjects were negatively correlated with Th1 cells. No correlation with Th17 cells was found. *In vitro* assays demonstrated this subset to inhibit autologous Th1 cell polarization, consistent with the negative immune regulation of this subset described in earlier studies [10]. Surprisingly, CD19^+^CD24^hi^CD38^hi^ B cells from PBC patients were positively correlated with Th1 cells and promoted Th1 cell differentiation in an *in vitro* coculture system. This result suggests that CD19^+^CD24^hi^CD38^hi^ B cells from PBC patients have lost their ability to negatively regulate immune cell function, but instead promote a positive immune response. Based on these findings and previous results, we propose a defective regulatory function for CD19^+^CD24^hi^CD38^hi^ B cells in PBC patients, which may partially be explained by Tim-1 downregulation and the skewing of proinflammatory cytokine polarization. Considering that CD19^+^CD24^hi^CD38^hi^ B cells from PBC patients displayed decreased IL-10 and increased IL-6 and IL-12 production when activated with anti-BCR and CpG, it is worth studying whether stimulated CD19^+^CD24^hi^CD38^hi^ B cells may acquire stronger Th1 cell regulation capacity *in vitro* costimulated with CD19^+^CD24^hi^CD38^hi^ B cells and T cell culture system.

This study was limited by the large amount of blood required for functional assays, which hampered the recruitment of patients. Further, there are no direct data demonstrating that lower levels of Tim-1 result in proinflammatory cytokine profile polarization of CD19^+^CD24^hi^CD38^hi^ B cells, promoting Th1 cell differentiation. In addition, only circulating and not local CD19^+^CD24^hi^CD38^hi^ B cells were evaluated.

In summary, we demonstrated for the first time that CD19^+^CD24^hi^CD38^hi^ B cells derived from PBC patients are not only increased in number but are also functionally abnormal. The CD19^+^CD24^hi^CD38^hi^ B cell subset cytokine polarization profile correlated with in vitro function and clinical status, which may contribute to the pathogenesis of PBC and the autoimmune process. Although further investigations are needed to define a precise role for CD19^+^CD24^hi^CD38^hi^ B cells, we propose that this subset is an inflammatory influence that supports Th1 cell differentiation. Further, therapeutic approaches directed against this subset may alleviate disease.

## Figures and Tables

**Table 1:** Clinical features and biochemical parameters of the PBC patients enrolled in this study.

	PBC	HC
Case	38	38
Gender (male/female)	10/28	10/28
Age (years)	62.4 ± 12.1	61 ± 15.5
AMA (positive/negative)	38/0	—
AMA-M_2_ (positive/negative)	38/0	—
ALP (U/L)	308.2 ± 225	85.6 ± 25.4
ALT (U/L)	133.1 ± 82.1	30.3 ± 19.8
AST (U/L)	108.1 ± 59.6	20.6 ± 14.2
ADA (U/L)	21.7 ± 12.1	13.7 ± 6.1
GGT (U/L)	320.6 ± 303.1	21.3 ± 3.2
TBIL (*μ*mol/L)	75.4 ± 62.1	16.4 ± 12.3
DBIL (*μ*mol/L)	60.5 ± 58.4	5.4 ± 3.3
TBA (*μ*mol/L)	105.8 ± 94.7	2.3 ± 4.1
IgM (g/L)	4.7 ± 1.2	1.1 ± 0.4
Clinical stage		
Noncirrhotic stage (*n*)	21	—
Cirrhotic stage (*n*)	12	—
Decompensated cirrhotic stage (*n*)	5	—

Age is expressed as the mean ± standard deviation; other data are expressed as the median and range. PBC: primary biliary cholangitis; HC: healthy controls. Normal values: ALT, 7-40 U/L; AST, 13-35 U/L; ADA, 0-25 U/L; GGT, 7-45 U/L; ALP, 50-135 U/L; TBIL, 2.1-22.3 *μ*mol/L; DBIL, 0-6.5 *μ*mol/L; TBA, 0-10 *μ*mol/L.

## Data Availability

The data used to support the findings of this study are available from the corresponding author upon request.

## Abbreviations

PBC: Primary biliary cholangitis
Breg: Regulatory B cells
AMA: Antimitochondrial antibody
Tim-1: T cell immunoglobulin mucin domain-1
PBMC: Peripheral blood mononuclear cells
TLR: Toll-like receptor
BCR: B cell receptor
HC: Healthy control
ALT: Alanine aminotransferase
AST: Aspartate aminotransferase
ADA: Adenosine deaminase
GGT: *γ*-Glutamyltransferase
ALP: Alkaline phosphatase
TBIL: Total bilirubin
DBIL: Direct bilirubin
TBA: Total biliary acid.

## Supplementary Materials

Supplementary Materials Supplementary Figure 1: further supplement result 3.2 in our manuscript, which is a complementary method for Ki-67 staining (manuscript line 173-175). Supplementary Figure 2: further supplement result 3.3 in our manuscript. CD19^+^CD24^+^CD38^+^ B cell in a correlation with the stage of PBC evaluated not only on the basis of a lab test but on the histology (manuscript line 184-185). Supplementary Figure 3: further supplement result 3.7 in our manuscript, which is a completed cross-linked coculture experiment between CD4^+^T cells from HC or PBC, with CD19^+^CD24^high^CD38^high^ B cells from HC or PBC (manuscript line 235-239).
