# Supplementary material for: CD19+CD24hiCD38hi B Cell Dysfunction in Primary Biliary Cholangitis
Source: Mediators Inflamm. 2020 Feb 10;2020:3019378. doi: 10.1155/2020/3019378 (PMC7035571; doi:10.1155/2020/3019378)

Supplementary Materials

Supplementary Figure 1: Circulating $CD19^{+}CD24^{hi}CD38^{hi}$ B subset proliferation were evaluated by CFSE staining. PBMC were isolated from PBC patients and HC and then labeled with CFSE with or without anti-BCR and CpG stimulation. (a) and (c), Representative flow cytometric histogram of CFSE level in unstimulated or stimulated $CD19^{+}CD24^{hi}CD38^{hi}$ B cells from PBC patients and HC subjects. (b) and (d), Statistical analysis of percentage of proliferation in unstimulated or stimulated $CD19^{+}CD24^{hi}CD38^{hi}$ B cells. *$P < 0.05$.

Supplementary Figure 2: Circulating $CD19^{+}CD24^{hi}CD38^{hi}$ B subset frequency in whole PBC disease stage. PBC patients were divided into noncirrhotic stage (n=21), cirrhotic stage (n=12), and decompensated cirrhotic stage (n=5), and the frequency of circulating $CD19^{+}CD24^{hi}CD38^{hi}$ B cells were assessed by flow cytometry of the three different disease stage. ns: P>0.05.

Supplementary Figure 3: $CD19^{+}CD24^{hi}CD38^{hi}$ B cells from PBC patients also promoted autologous Th1 cell differentiation. $CD19^{+}CD24^{hi}CD38^{hi}$ B cells from PBC patients and HC subjects were sorted by flow cytometry and then co-cultured with PBC CD^{+}T cells for 6 D, and Th1 cell frequency was measured by flow cytometry. The data are representative of three separate experiments. *$P < 0.05$.

Supplementary Figure 1:

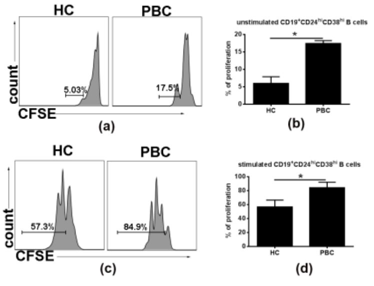

Supplementary Figure 2:

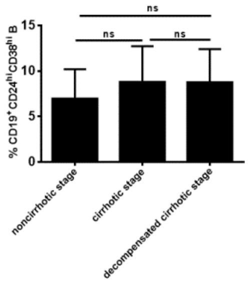

Supplementary Figure 3:

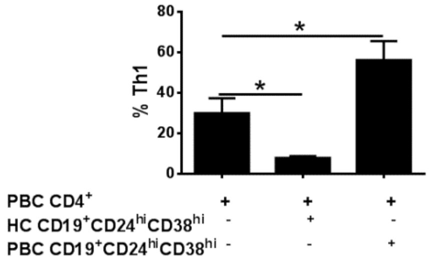

Supplement: Supplementary Materials — Supplementary Figure 1: further supplement result 3.2 in our manuscript, which is a complementary method for Ki-67 staining (manuscript line 173-175). Supplementary Figure 2: further supplement result 3.3 in our manuscript. CD19+CD24+CD38+ B cell in a correlation with the stage of PBC evaluated not only on the basis of a lab test but on the histology (manuscript line 184-185). Supplementary Figure 3: further supplement result 3.7 in our manuscript, which is a completed cross-linked coculture experiment between CD4+T cells from HC or PBC, with CD19+CD24highCD38high B cells from HC or PBC (manuscript line 235-239). [file 3019378.f1.pdf]
